# Interrelation of chemerin and TNF-α with mtDNA copy number in adipose tissues and blood cells in obese patients with and without type 2 diabetes

**DOI:** 10.1186/s12920-019-0485-8

**Published:** 2019-03-13

**Authors:** Daria Skuratovskaia, Pavel Zatolokin, Maria Vulf, Ilia Mazunin, Larisa Litvinova

**Affiliations:** 1 Immanuel Kant Baltic Federal University, Russian Federation Kaliningrad, Gaidara 6 st, Russia; 2 Department of Reconstructive and Endoscopic Surgery, Kaliningrad Regional Hospital Kaliningrad, Russia

## Abstract

**Background:**

Inflammatory response plays a key role in the development of insulin resistance (IR) in obesity. Oxidative stress triggers the replication of the mitochondrial genome and division of the organelle. The purpose of this study was to identify the relationship of chemerin and TNF-α with mitochondrial DNA (mtDNA) copy number in subcutaneous adipose tissue (SAT) and visceral adipose tissue (mesentery of the small intestine (Mes), greater omentum (GO) and blood mononuclear cells (MNCs)) in patients with obesity with/without type 2 diabetes mellitus (T2DM).

**Methods:**

The study included 142 obese patients and 34 healthy donors. The samples used for the study were peripheral venous blood (MNCs) and ATs (SAT, Mes and GO). The measurement of mtDNA copy number was done by droplet digital PCR. Quantitative determination of insulin, adiponectin, TNF-α and chemerin in serum/plasma was performed by flow-through fluorometry and commercial ELISA kit. Statistical analysis and graphs were obtained in R Statistical Software (version 3.3.1).

**Results:**

The increase in body mass index (BMI) was accompanied by an increase in TNF-α production, an increase in mtDNA copy number in SAT and a decrease in mtDNA copy number in MNCs. The negative association between plasma chemerin and mtDNA copy number in the Mes, as well as between mtDNA copy number and chemerin expression in the Mes, in the group with BMI > 40 kg/m^2^ without T2DM demonstrates the protective effect of chemerin against the development of IR via the regulation of mtDNA copy number in adipose tissues.

**Conclusions:**

We thus speculated the existence of a compensatory mechanism in which leads to the increased number of mtDNA copies under the influence of proinflammatory factors. Based on the obtained data, we propose that reducing mtDNA copy number in cases of morbid obesity without T2DM has a positive effect on carbohydrate metabolism, which may help maintain glucose levels within reference values.

*Obesity, type 2 diabetes, mtDNA, cytokines, TNF-a, chemerin.*

## Background

Inflammatory response plays a key role in the development of insulin resistance (IR) in obesity [1, 2]. The initial pathogenetic factors in the development of IR in obesity are not fully understood. Mitochondria are involved in the process of lipolysis in adipocytes and are the main source of ATP in cells [3]. The malfunctioning of mitochondria leads to an energy crisis in insulin-dependent tissues and forms the foundation for the formation of IR [4, 5]. The high content of free fatty acids induces mitochondrial DNA (mtDNA) damage and causes the production of endoplasmic reticulum stress markers, protein degradation and apoptosis, contributing to increased oxidative stress in skeletal muscle cells and adipose tissues (ATs) [6]. Oxidative stress triggers the replication of the mitochondrial genome and division of the organelle [7, 8].

Some authors have studied the role of chemerin in regulating the function of mitochondria [8]. Сhemerin participates in processes of lipogenesis, metabolism, angiogenesis, inflammation, and proliferation and differentiation of adipocytes by regulating the expression of respective genes [9]. The mechanism underlying the effect chemerin has on mitochondrial function is mostly unknown. Xie Q. et al. (2015) have shown that chemerin-induced mitochondrial dysfunction contributes to a reduction in mitochondrial biogenesis and an increase in mitochondrial autophagy. The dual role of chemerin in inflammatory processes and metabolism may provide a link between chronic inflammation and energy metabolism in obesity, as well as the complications associated with this condition.

The purpose of this study was to define the relationship of chemerin and TNF-α with mtDNA copy number in subcutaneous adipose tissue (SAT) and visceral adipose tissue (VAT; mesentery of the small intestine (Mes), greater omentum (GO) and blood mononuclear cells (MNCs)) in obese patients with/without type 2 diabetes mellitus (T2DM).

## Materials and methods

### Study subjects

The study included 142 obese patients treated at the Regional Clinical Hospital of the Kaliningrad Region (Table 1). The control group consisted of 34 healthy donors with normal anthropometric and biochemical parameters of carbohydrate and lipid metabolism.

**Table 1:** General characteristics of study groups

	Control group	Pre-obese	grade I obesity		grade II obesity		grade III obesity	
			Without T2DM	With T2DM	Without T2DM	With T2DM	Without T2DM	With T2DM
Groups	1 *n* = 34	2 *n* = 24	3 *n* = 10	4 *n* = 18	5 n = 10	6 *n* = 19	7 *n* = 15	8 *n* = 46
BMI, kg/m^2^	18,5 – 24,9	25–29,9	30–34,9	30–34,9	35–39,9	35–39,9	› 40	› 40
Age, year	37.4 ± 10.3	46 ± 9.8	43.9 ± 4.9	45 ± 7,9	43.1 ± 7.5	48.5 ± 7.2	47.6 ± 9.6	48.3 ± 7.7

Patients with abdominal obesity were ranked by the degree of obesity into the following groups: 2 - BMI = 24.9–29.9 kg/m^2^; 3 and 4 - BMI = 30.0–34.9 kg/m^2^; 5 and 6 - BMI = 35–39.9 kg/m^2^; and 7 and 8 - BMI > 40 kg/m^2^; patients were ranked by the state of carbohydrate metabolism as follows: patients with T2DM (groups 4, 6, and 8) and patients without T2DM (groups 2, 3, 5, and 7).

- Patients with pre-obesity were included in the second group.
- The third and fourth groups comprised patients with grade I obesity stratified by the state of carbohydrate metabolism.
- The fifth and sixth groups comprised patients with grade II obesity stratified by the state of carbohydrate metabolism.
- Patients with BMI > 40 kg/m^2^ were in the seventh and eighth groups with grade III obesity and stratified by the state of carbohydrate metabolism.

All groups were comparable age- and sex-wise.

The samples used for the study were peripheral venous blood (MNCs) and ATs (SAT, Mes and GO).

The presence of obesity and T2DM was established on the basis of a detailed clinical and instrumental examination in a specialized hospital, guided by the World Health Organization (1999–2013) criteria for diagnosing diabetes and other types of hyperglycemia [10]. Informed consent was signed by all patients. Verification of the diagnosis and recruitment of patients into the study groups was carried out at the Department of Reconstructive and Plastic Surgery on the basis of regional clinical hospital in Kaliningrad.

Permission to conduct the study was obtained from the local ethics committee (Minute No. 4 of the meeting of the Local Ethics Committee at the Innovation Park of the Immanuel Kant Baltic Federal University, dated October 23, 2013).

### Droplet digital PCR

DNA extraction from MNCs and ATs (SAT, Mes and GO) was carried out using commercial QIAamp DNA Mini Kit according to the manufacturer’s protocol (Qiagen, USA).

The DNA concentration in the samples was measured with an Implen NanoPhotometer spectrophotometer at a wavelength of 260 nm. The quality of extracted DNA was determined from the absorption ratio at 260 and 280 nm (A260/280 > 1.8).

mtDNA copy number was determined by ddPCR. For this, the extracted DNA samples were pretreated with ApaI restriction endonuclease (New England BioLabs, Ipswich, MA, USA) (1 μl (10 U) ApaI, 5 μl 10x CutSmart® buffer, and ~ 30 ng DNA sample), and then the reaction mixture was incubated for 3–4 h at 37 °C. The components of the ddPCR itself included a commercial PCR mixture, selected fluorescent TaqMan probes, and oligonucleotide primers (Table 2). Oligonucleotide primers and TaqMan probes labeled with fluorescent HEX and FAM were selected to amplify the corresponding portions of the mitochondrial gene *ND1* (encoding subunit 1 of the NADH dehydrogenase complex) and the single-copy nuclear gene *RPP30* (encoding the P/MRP subunit P30 subunit); the sequences are indicated in Table 2. The composition of the multiplex mixture of ddPCR included 10 μl of commercial 2x ddPCR Master Mix (Bio-Rad, Pleasanton, USA), 1 μl of each primer (final concentration 450 nM), FAM- and HEX-labeled TaqMan probes (the final concentration of each probe in the mixture was 125 nM) and 8 μl of DNA sample. The prepared reaction mixture was loaded into an automatic droplet generator (Bio-Rad, Pleasanton, USA), where it was emulsified into approximately 15–20 thousand drops, each approximately one nanoliter in volume.

**Table 2:** Sequence of primers and Taqman probes

Gene	Primer (5′-3′)	Product length
Mitochondrial gene ND1		
ND1_f	ccctaaaacccgccacatct	65
ND1_r	gagcgatggtgagagctaaggt	
ND1_probe	HEX-ccatcaccctctacatcaccgccc-BHQ1	
Nuclear gene P30		
RPP30_f	gatttggacctgcgagcg	62
RPP30_r	gcggctgtctccacaagt	
RPP30_probe	FAM-ctgacctgaaggctct-BHQ1	
TNF-а		
TNFA_f	ccctcaacctcttctggctcaa	97
TNFA_r	ccaggtttcgaagtggtggtct	
RARRES2 (Chemerin)		
RARRES2_f	tgaggacccccacagcttct	92
RARRES2_r	aggcaccacgcatctcagtg	
ADIPOQ (Adiponectin)		
ADIPOQ_f	tccccaacatgcccattcgct	97
ADIPOQ_r	agcccaggaatgttgcagtgga	
B2M (Reference gene)		
B2M_f	cctgccgtgtgaaccatgtg	70
B2M_r	gctgcttacatgtctcgatccca	

In the next step, the emulsified ddPCR mixture was transferred to standard 96-well plates and amplified in a thermocycler (Bio-Rad T100 thermal cycler) according to the following temperature protocol: 95 °C for 10 min, 40 cycles at 94 °C for 30 s and 53 °C for 60 s and a final cycle at 98 °C for 10 min. After the amplification reaction, strength of fluorescence was measured using the QX200 Droplet Reader (Bio-Rad, Pleasanton, CA) which operates similarly to a flow cytometer. The data were analyzed in the QuantaSoft program suite (version 1.7.4.0917). The absolute number of copies of mtDNA per cell was calculated using methods outlined below.

### Quantitative PCR

Total RNA from homogenized AT biopsies was isolated using ExtractRNA kit (Eurogen, Russia). The resulting RNA was dissolved in 30 μl of RNAse- and DNAse-free water. The purity and concentration of isolated RNA were determined using a spectrophotometer (Nanovue Plus, GE Healthcare Bio-Sciences, Sweden). The quality of total RNA was determined by the RNA Integrity Number (RIN). Reverse transcription was performed using (dT) 23 (Beagle, Russia) and LV-revertase (Eurogen, Russia). To determine the levels of relative gene expression qPCR was performed using the qPCR mix-HS SYBR reagents (Eurogen, Russia). Approximately 4 μl cDNA was used as template, and *B2M* was used as the reference gene. The primer sequences are shown in Table 2.

### Blood chemistry

Parameters of carbohydrate and lipid metabolism were studied on an automatic biochemical analyzer CA-180 (Furuno Electric Co., Ltd., Japan).

### Enzyme-linked immunosorbent assay

The concentration of molecules (TNF-α and chemerin) was measured in serum/plasma using a sandwich enzyme-linked immunosorbent assay (ELISA) (Vector-Best kits, Russia The range of measured concentrations is 0–1000 pg / ml, sensitivity - 2 pg / ml; BIO Vendor, Czech Republic, the range of measured concentrations is 0.25–8 ng / ml, sensitivity 0.1 ng / ml).

### Flow cytometry

Quantitative determination of insulin and adiponectin in plasma was performed by flow-through fluorometry using commercial test systems (Bio-Rad, USA) on a two-beam laser automated analyzer (BioPlex® 200 Systems, Bio-Rad, USA) and BioPlex Manager (Bio-Rad, USA).

### Statistical analysis

Verification of the normality of quantative indicator distribution was carried out using the Kolmogorov-Smirnov and the Shapiro-Wilk tests. Because the investigated samples fit under normal distribution, the hypothesis of the equality of the mean sample values was verified using Student’s t-tests. To assess the significance of differences between independent quantitative samples that do not follow a normal distribution law, non-parametric Kruskal-Wallis test was used. For detecting statistically significant differences between groups, a pairwise analysis was performed using the Mann-Whitney test. Differences were considered significant at the level of *p* < 0.05.

Correlations between the studied indices were determined using the Spearman correlation analysis and linear regression. For the analysis of an adequate linear regression model, the following regression residues were considered: the lack of autocorrelation of residues (Durbin-Watson test, *p*-values> 0.05) and its normal distribution and the consistency of the dispersion residues (heteroscedasticity test, p-values< 0.05 were considered significant). Statistical analysis and graphs were obtained in R Statistical Software (version 3.3.1) and Statistica 10.

## Results

### Plasma level of components of carbohydrate metabolism, proinflammatory mediators (TNF-α and CRP) and adipokines (chemerin and adiponectin)

The level of glucose and the HOMA-IR was higher in obese patients with T2DM (with grade I, II, and III obesity) than in patients without T2DM and control individuals (*p* < 0.05) (Table 3). The plasma level of proinflammatory molecules (C-reactive protein (CRP) and TNF-α) increased along with BMI (Table 3).

**Table 3:** Change in plasma levels of adiponectin, chimerine, TNF-a and IL-6 in the study groups of patients

	Control group	Pre-obese	Patients with obesity					
BMI, kg/m^2^	18,5 – 24,9	25–29,9	grade I obesity (30–34,9)		grade II obesity (35–39,9)		grade III obesity (› 40)	
			Without T2DM	With T2DM	Without T2DM	With T2DM	Without T2DM	With T2DM
Groups	1	2	3	4	5	6	7	8
TNA-a pg/ml	2,03 (1,46 – 3,72)	1,92 (1,43 – 2,15) p_1–2_ = 0,47	4,07 (3,37 – 8,56) p_1–3_ = 0,03* p_2–3_ < 0,01*	20,16 (15,5 – 22,08) p_1–4_ < 0,01* p_2–4_ < 0,01* p_3–4_ < 0,01*	10,82 (5,68 – 13,89) p_1–5_ < 0,01* p_2–5_ < 0,01* p_3–5_ = 0,05 p_4–5_ < 0,01*	28,21 (19,2 – 40,19) p_1–6_ < 0,01* p_2–6_ < 0,01* p_3–6_ < 0,01* p_4–6_ = 0,02* p_5–6_ < 0,01*	14,71 (9,63 – 17,3) p_1–7_ < 0,01* p_2–7_ < 0,01* p_3–7_ < 0,01* p_4–7_ = 0,69 p_5–7_ = 0,06 p_6–7_ < 0,01*	32,86 (29,9 –34,16) p_1–8_ < 0,01* p_2–8_ < 0,01* p_3–8_ < 0,01* p_4–8_ < 0,01* p_5–8_ < 0,01* p_6–8_ = 0,84 p_7–8_ < 0,01*
Chemerin ng/ml	128 (117–147)	128,5 (103–157,5) p_1–2_ = 0,99	166 (147–172) p_1–3_ = 0,04* p_2–3_ = 0,03*	170,5 (151,5 – 179,5) p_1–4_ = 0,04* p_2–4_ = 0,04* p_3–4_ = 0,44	169 (148–177) p_1–5_ = 0,01* p_2–5_ = 0,03* p_3–5_ = 0,47 p_4–5_ = 0,93	150 (130,5 – 175,5) p_1–6_ = 0,10 p_2–6_ = 0,17 p_3–6_ = 0,75 p_4–6_ = 0,38 p_5–6_ = 0,53	190 (180–196) p_1–7_ < 0,01* p_2–7_ < 0,01* p_3–7_ = 0,04* p_4–7_ = 0,06 p_5–7_ = 0,049* p_6–7_ = 0,02*	143 (134–158) p_1–8_ = 0,05 p_2–8_ = 0,18 p_3–8_ = 0,26 p_4–8_ = 0,28 p_5–8_ = 0,16 p_6–8_ = 0,75 p_7–8_ < 0,01*
Adiponectin mkg/ml	3,54 (2,59 – 4,27)	1,74 (1,29 – 4,16) p_1–2_ = 0,18	2,27 (1,8 – 4,19) p_1–3_ = 0,25 p_2–3_ = 0,32	3,81 (2,87 – 4,14) p_1–4_ = 0,84 p_2–4_ = 0,15 p_3–4_ = 0,25	4,5 (1,9 – 5,48) p_1–5_ = 0,61 p_2–5_ = 0,07 p_3–5_ = 0,27 p_4–5_ = 0,99	3,67 (2,92–5,18) p_1–6_ = 0,61 p_2–6_ = 0,07 p_3–6_ = 0,28 p_4–6_ = 0,99 p_5–6_ = 0,98	4,44 (2,87–5,21) p_1–7_ = 0,39 p_2–7_ = 0,03* p_3–7_ = 0,15 p_4–7_ = 0,70 p_5–7_ = 0,69 p_6–7_ = 0,69	3,86 (1,85 – 4,71) p_1–8_ = 0,85 p_2–8_ = 0,11 p_3–8_ = 0,37 p_4–8_ = 0,88 p_5–8_ = 0,65 p_6–8_ = 0,65 p_7–8_ = 0,49
CRP mmol/l	4,41 (3,12–5,38)	1,8 (1,51 – 4,78) p_1–2_ = 0,058	1,9 (1,03 – 2,83)	7,72 (4,6 – 8,88)	7,6 (2,2 – 13,4)	5,7 (5,52 – 9,24) p_2–6_ = 0,036*	13,4 (9,9 – 15,99) p_1–7_ = 0,004* p_2–7_ = 0,0017* p_3–7_ = 0,01*	8,95 (5,04 – 14,34) p_1–8_ = 0,045* p_2–8_ = 0,004*
Glucose mmol/l	3,75 (1,7 – 5,24)	5,18 (4,98 – 5,34) p_1–2_ = 0,01*	5,6 (5,2 – 5,83) p_1–3_ = 0,02*	10,74 (9,74 – 10,88) p_1–4_ = 0,003*	5,8 (5,6 – 6,3) p_1–5_ = 0,002*	8,1 (6,7 – 11,8) p_1–6_ = 0,007*	5,9 (5,5 – 6,6) p_1–7_ = 0,01*	7,1 (5,8 – 9,6) p_1–8_ = 0,005*
Insulin ng/ml	47,09 (27,16 - 87,18)	45,06 (16,93 - 76,40)	60,62 (30,77 - 90,83)	84,67 (42,03 - 135,37)	176 (124–248) *p* = 0,01	139 (119–476) *p* = 0,035	216 (94,38 - 371,6) *p* = 0,033	304 (244–616) *p* = 0,001

The serum level of CRP was 8.95 (5.04–14.34) mmol/l in the group with T2DM (BMI > 40 kg/m^2^) and 13.4 (9.9–15.99) mmol/l in the group without T2DM (BMI > 40 kg/m^2^), exceeding the values in the control group of 4.41 (3.12–5.38) mmol/l (*p* < 0.05).

The plasma level of TNF-α in obese patient with T2DM exceeded that in patients without T2DM and control individuals (*p* < 0.05).

**Fig. 1:**
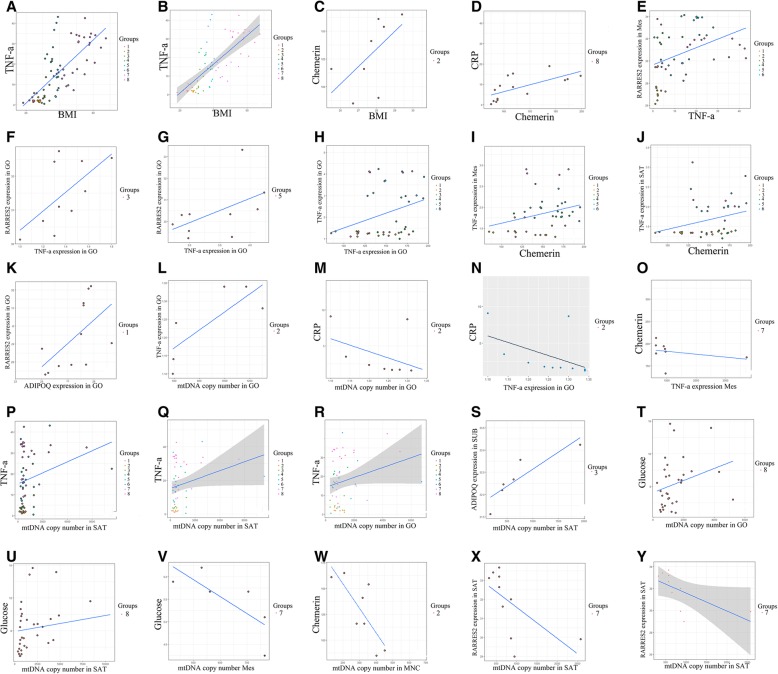
Correlation and regression relationships between studied metabolites.Note: Correlation - **a**, **c**, **d**, **e**, **f**, **g**, **h**, **i**, **j**, **k**, **l**, **m**, **n**, **o**, **p**, **s**, **t**, **u**, **v**, **w**, **x**; Regression – **b**, **q**, **r**, **y**

Correlation (*r* = 0.76, *p* < 0.001) and regression (r^2^ = 70, *p* < 0.001) analyses revealed an association between the concentration of TNF-α and BMI in all groups (*p* < 0.05) (Fig. 1a, b).

Plasma level of chemerin increased in patients without T2DM (with grade I, II and III obesity); however, the plasma level of chemerin in patients with T2DM did not differ from that in control individuals (with grade II and III obesity).

The plasma level of chemerin in patients without T2DM (with grade I, II and III obesity) and in patients with T2DM (with grade I obesity) exceeded the control values and values in pre-obese individuals (*p* < 0.05). In pre-obese individuals, the plasma level of chemerin was positively correlated with BMI (*r* = 0.71, *p* < 0.05) (Fig. 1c).

Positive correlations between plasma level of chemerin and CRP were observed in patients with T2DM (*r* = 0.76, *p* < 0.01) (Fig. 1d).

Furthermore, the plasma level of adiponectin in patients without T2DM (III degree of obesity) was higher than in pre-obese patients (4.44 (2.87–5.21) μg/ml vs. 1.74 (1.29–4.16) μg/ml).

### Expression of mediators (TNF-α, RARRES2, and ADIPOQ) in different ATs

**Fig. 2:**
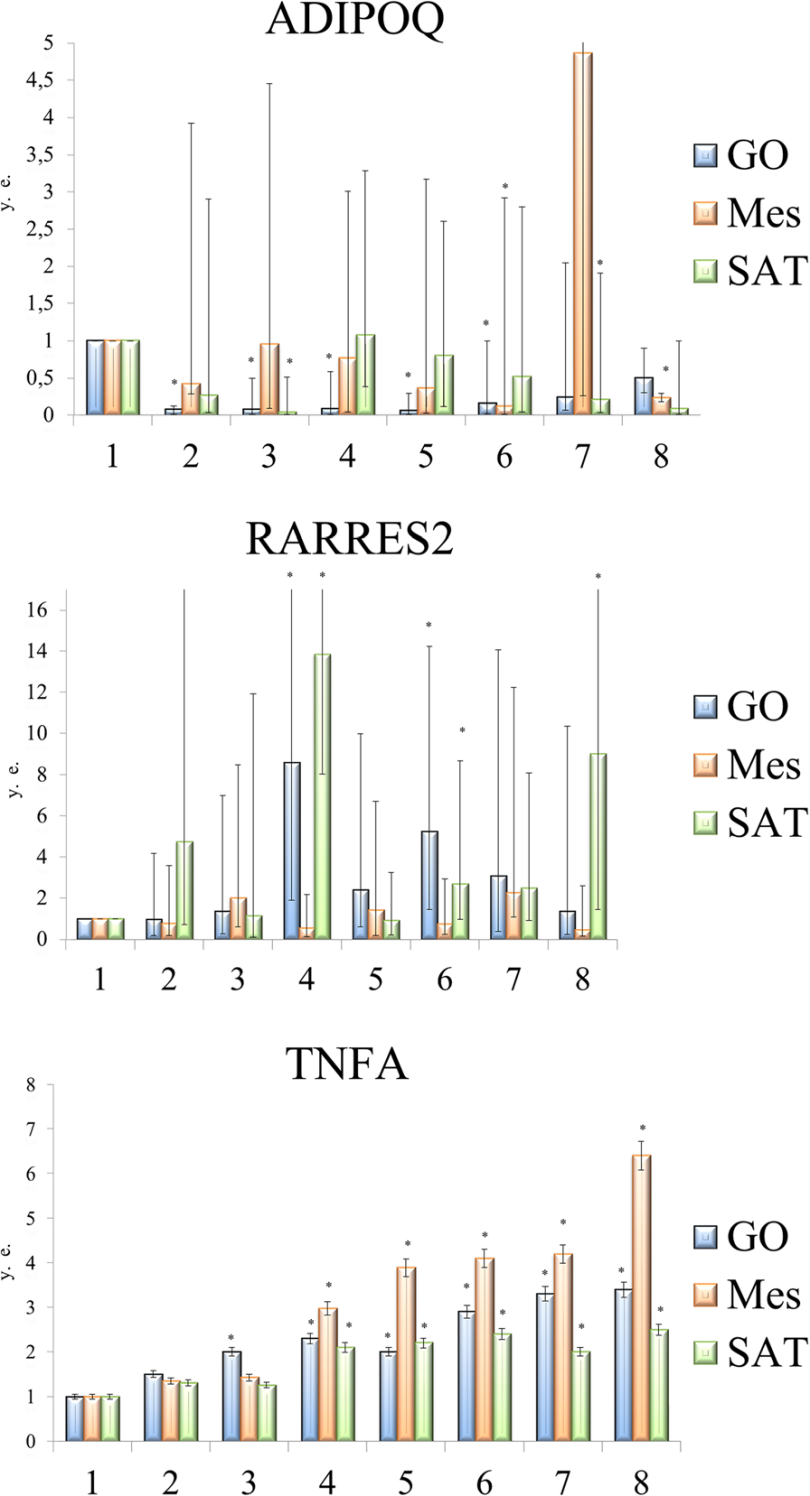
Changes in the level of relative expression in adipose tissue of different locations in obese patients associated with and without type 2 diabetes

The expression of the *TNF-α* gene was increased in all fat stores relative to that in control. The highest level of *TNF-α* was detected VAT (GO and Mes) (Fig. 2a). The expression of the *RARRES2* gene (encoding chemerin) increased in SAT in patients with T2DM with respect to that in control individuals. In patients with T2DM (with grade II and III obesity), the expression of the *RARRES2* gene increased in the GO and SAT.

Interestingly, the expression of *TNF-α* and *RARRES2* genes in the GO and SAT in patients with T2DM was higher than in patients without T2DM (Fig. 2b).

The plasma level of TNF-α was positively correlated with the expression of the *RARRES2* gene in the Mes in all groups with BMI < 40 kg/m^2^ (Fig. 1e). The expression level of the *TNF-α* gene was positively correlated with the expression level of the *RARRES2* gene in the GO in patients with T2DM (with grade I and II obesity) (*r* = 0.66, *p* < 0.05 and *r* = 0.67, *p* < 0.05, respectively) (Fig. 1f, g).

Positive correlations were also observed between the expression of the *TNF-α* gene in the *GO* (*r* = 0.34, *p* < 0.05), Mes (*r* = 0.34, *p* < 0.05) and SAT (*r* = 0.37, *p* < 0.05) and the plasma level of chemerin in all groups with BMI < 40 kg/m^2^ (Fig. 1h, i, j).

In general, the expression of the *ADIPOQ* gene in the Mes tended to increase in patients without T2DM and tended to decrease in patients with T2DM. The *ADIPOQ* gene is expressed predominantly in the SAT in patients with T2DM and in the GO in patients without T2DM (Fig. 2c). Under physiological conditions, chemerin may have anti-inflammatory properties within the visceral fluid, and this was confirmed by positive correlations between the expression of *ADIPOQ* and *RARRES2* genes in the GO (*r* = 0.7, *p* < 0.05) in the control group (Fig. 1k).

### mtDNA copy number in different ATs and MNCs

In general, there was an increase in mtDNA copy number in different ATs with an increase in BMI (Table 4).

**Table 4:** Change in the number of copies of mtDNA in the study groups of patients in tissues of different locations (Copies per cell)

	Control group	Pre-obese	Patients with obesity					
BMI, kg/m^2^	18,5 – 24,9	25–29,9	grade I obesity (30–34,9)		grade II obesity (35–39,9)		grade III obesity (› 40)	
			Without T2DM	With T2DM	Without T2DM	With T2DM	Without T2DM	With T2DM
Groups	1	2	3	4	5	6	7	8
GO	855 (744–993)	997 (592–1275) p_1–2_ = 0,76	703 (298–1293) p_1–3_ = 0,73 p_2–3_ = 0,66	590 (541–633) p_1–4_ = 0,18 p_2–4_ = 0,19 p_3–4_ = 0,81	947 (503–1758) p_1–5_ = 0,35 p_2–5_ = 0,57 p_3–5_ = 0,20 p_4–5_ = 0,20	1619 (963–2228) p_1–6_ = 0,08 p_2–6_ = 0,29 p_3–6_ = 0,10 p_4–6_ = 0,01* p_5–6_ = 0,46	733 (465–834) p_1–7_ = 0,63 p_2–7_ = 0,33 p_3–7_ = 0,62 p_4–7_ = 0,56 p_5–7_ = 0,24 p_6–7_ = 0,02*	1080 (769–1663) p_1–8_ = 0,28 p_2–8_ = 0,33 p_3–8_ = 0,09 p_4–8_ = 0,03* p_5–8_ = 0,78 p_6–8_ = 0,15 p_7–8_ = 0,03*
Mes	791 (637–1031)	841 (549–1160) p_1–2_ = 0,74	632 (530–765) p_1–3_ = 0,50 p_2–3_ = 0,20	993,500 (615–2257) p_1–4_ = 0,35 p_2–4_ = 0,83 p_3–4_ = 0,20	1132(1044–1325) p_1–5_ = 0,06 p_2–5_ = 0,08 p_3–5_ = 0,01* p_4–5_ = 0,56	1630 (1360–1835) p_1–6_ = 0,08 p_2–6_ = 0,01* p_3–6_ < 0,01* p_4–6_ = 0,28 p_5–6_ = 0,06	900 (637–960) p_1–7_ = 0,12 p_2–7_ = 0,91 p_3–7_ = 0,04* p_4–7_ = 0,73 p_5–7_ = 0,02* p_6–7_ < 0,01*	925 (711–1653) p_1–8_ = 0,07 p_2–8_ = 0,25 p_3–8_ = 0,01* p_4–8_ = 0,98 p_5–8_ = 0,50 p_6–8_ = 0,07 p_7–8_ = 0,14
SAT	462 (316–820)	627 (569,5–911) p_1–2_ = 0,07	527 (402–759) p_1–3_ = 0,71 p_2–3_ = 0,36	1206,5 (533–1820) p_1–4_ = 0,14 p_2–4_ = 0,46 p_3–4_ = 0,33	460 (346–1909) p_1–5_ = 0,56 p_2–5_ = 0,29 p_3–5_ = 0,96 p_4–5_ = 0,57	931 (642–3855) p_1–6_ = 0,07 p_2–6_ = 0,22 p_3–6_ = 0,15 p_4–6_ = 0,70 p_5–6_ = 0,17	613 (568–862) p_1–7_ = 0,09 p_2–7_ = 0,71 p_3–7_ = 0,45 p_4–7_ = 0,39 p_5–7_ = 0,37 p_6–7_ = 0,13	1059 (635–2484) p_1–8_ < 0,01* p_2–8_ = 0,13 p_3–8_ = 0,03* p_4–8_ = 0,52 p_5–8_ = 0,02* p_6–8_ = 0,96 p_7–8_ = 0,01*
MNC	224,5 (159,7 – 315,5)	323(180–414) p_1–2_ = 0,06	149 (126–242) p_1–3_ = 0,27 p_2–3_ = 0,02*	296(163–429) p_1–4_ = 0,56 p_2–4_ = 0,87 p_3–4_ = 0,18	145 (139–209) p_1–5_ = 0,17 p_2–5_ = 0,01* p_3–5_ = 0,90 p_4–5_ = 0,23	102 (89–141) p_1–6_ = 0,02* p_2–6_ < 0,01* p_3–6_ = 0,13 p_4–6_ = 0,16 p_5–6_ = 0,06	177	156 (113–229) p_1–8_ = 0,13 p_2–8_ < 0,01* p_3–8_ = 0,96 p_4–8_ = 0,21 p_5–8_ = 0,98 p_6–8_ = 0,06 p_7–8_ = 0,47

mtDNA copy number in the GO was 1619 (963–2228) in patients with T2DM (with grade II obesity), which was higher than the reference value and higher than the value in patients with T2DM (with grade I obesity) (590 (541–633) (*p* < 0,05). mtDNA copy number in the GO was 2-times higher (1080 (769–1663)) in patients with T2DM (with grade III obesity) than in patients with grade I obesity 590 (541–633) (*p* < 0,05). Furthermore, mtDNA copy number in the GO in patients with T2DM (with grade III obesity) was higher than that in patients without T2DM (with grade III obesity) (1080 (769–1663) vs. 733 (465–834)) (*p* < 0,05).

A positive correlation was observed between mtDNA copy number in the GO with the expression of the *TNF-α* gene in the GO in pre-obese individuals (*r* = 0.81, *p* < 0.05), and a negative correlation was observed between these indices and the plasma level of CRP (*r* = − 0.8, *r* = − 0.79, *p* < 0.05) (Fig. 1l, m, n).

The number of copies of mtDNA in the Mes (1630 (1360–1835)) in patients with T2DM (with grade II obesity) was higher than in pre-obese individuals 841 (549–11,160)) (*p* < 0,05). mtDNA copy number in the Mes in patients with grade III obesity was lower than that in patients with grade II obesity regardless of the presence/absence of T2DM. mtDNA copy number in the Mes was negatively related to the plasma level of chemerin in patients without T2DM (with grade III obesity) (*r* = − 0.72, *p* < 0.05) (Fig. 1o).

Moreover, mtDNA copy number in the SAT was higher in patients with T2DM than in patients without T2DM. mtDNA copy number in the SAT was 1059 (635–2484) in patients with T2DM (with grade III obesity, respectively) and exceeded the control value of 462 (316–820). Furthermore, mtDNA copy number in SAT (1059 (635–2484)) in patients with T2DM was higher than that in patients without T2DM (with grade III obesity) (Table 4).

Positive correlation and regression were observed between mtDNA copy number in the SAT and the plasma level of TNF-α (*r* = 0.34, r^2^ = 24.7, *p* < 0.05) and mtDNA copy number in the GO (r^2^ = 0.27, *p* < 0.05) in all study groups (Fig. 1p, q, r).

Positive correlations were also observed between mtDNA copy number and the expression level of the *ADIPOQ* gene (*r* = 1.0, *p* < 0.01) in SAT in patients without T2DM (with grade I obesity) (Fig. 1s).

mtDNA copy number in the GO and SAT was positively correlated with serum glucose level (*r* = 0.39, *r* = 0.38, *p* < 0.05) in patients with T2DM (BMI > 40 kg/m^2^) (Fig. 1t, u). In contrast, mtDNA copy number in the Mes was not correlated with blood glucose level (*r* = − 0.9, *p* < 0.05) in patients without T2DM (with grade I obesity) (Fig. 1v).

mtDNA copy number in the MNCs was higher in the pre-obese group (323 (180–414)) than in all other study groups (*p* < 0.05). However, mtDNA copy number in MNCs was lower in patients with T2DM (with grade II obesity) than in patients without T2DM (with grade II obesity) and in pre-obese and control individuals (Table 4). mtDNA copy number in MNCs was also negatively correlated with the plasma level of chemerin in pre-obese individuals (*r* = − 0.77, *p* < 0.05) (Fig. 1w). However, mtDNA copy number in the SAT was negatively associated with the expression of the *RARRES2* gene in patients without T2DM (with grade III obesity) (*r* = − 0.84, *p* < 0.05; r^2^ = − 0.69, *p* < 0.05) (Fig. 1x, y).

## Discussion

Hypertrophy of adipocytes contributes to the development of hypoxia in AT during obesity and the release of free fatty acids during lipolysis [11]. In ATs, immune cells and macrophages are recruited by inflammatory mediators, such as HIF-1 and MCP-1 [12]. Macrophages ingest necrotic adipocytes and produce chemokines and proinflammatory mediators, including chemerin and TNF-α [12, 13]. Thus, a positive inflammatory feedback loop is formed.

In all obese patients, with or without T2DM, the expression level of the *TNF-α* gene in the GO, Mes, and SAT and the plasma level of TNF-α increase along with the BMI. Correlation and regression analyses revealed an association between the BMI and the plasma level of TNF-α in all study groups (Fig. 1a, b).

In our study, the change in metabolic parameters at the earliest stage of obesity in pre-obese patients was particularly interesting (BMI = 25–29.9 kg/m^2^). Positive correlation was observed between mtDNA copy number with the expression of the *TNF-α* gene in the GO in pre-obese individuals (*r* = 0.81, *p* < 0.05), and a negative correlation was observed between these indices and the plasma level of CRP (Fig. 1l, m, n). CRP characterizes a systemic inflammatory response [14]. The associations revealed by us indicate the local course of inflammation in the AT at early stages of obesity.

Increasing BMI is accompanied by an increase in TNF-α production, which ultimately leads to an increase in mtDNA copy number [1, 8]. Mitochondria generate superoxide, hydrogen peroxide and hydroxyl radicals, contributing to damage protein, DNA, RNA and lipids. Oxidative stress promotes nuclease activity against mtDNA, and this leads to the accumulation of mtDNA fragments [13, 15]. The disruption of the native structure of mtDNA in IR is reflected by a decrease in the efficiency of oxidative phosphorylation and ATP synthesis [4].

We speculate the existence of a compensatory mechanism in which mtDNA copy number increases under the influence of proinflammatory factors. This hypothesis was confirmed by positive correlations of the plasma level of TNF-α with mtDNA copy number in the SAT and GO in all study groups (Fig. 1p, q, r).

mtDNA copy number in the VAT (GO and MES), regardless of the state of carbohydrate metabolism, increased in patients with grade II and III obesity in relation to that in patients with grade I obesity. However, in patients with morbid obesity (BMI > 40 kg/m^2^) with and without T2DM, mtDNA copy number in the Mes was reduced by a factor of 2 compared to that in patients with grade II obesity and was comparable to that in control individuals. This correlation was not observed in other tissues (GO, SAT or MNCs).

A reduction in the number of copies of mtDNA in the GO in patients with morbid obesity in comparison with that in patients with grade I and II obesity indicates a change in the energy metabolism in this fat depot. When the TNF signaling pathway is activated, in addition to the initiation of mitochondrial division by DNM1L, the activation of caspases takes place, initiating the cell death programs apoptosis and necrosis [8, 16, 17]. Studies in animal models with obesity have shown that a change in mitochondrial biogenesis is a possible response to prolonged exposure to reactive oxygen species (ROS) – in the first months the amount of mtDNA increases to compensate for the damage induced by oxidation, and under further stress, intracellular depletion occurs beyond the recovery capacity of the cell, and the amount mtDNA drops sharply [18].

mtDNA dysfunction is less associated with damage to single copies of mtDNA but more so with a dysregulation of mtDNA copy number in the cell [19]. Recently, Ye J. (2013) proposed a concept regarding the relationship between energy metabolism in mitochondria and IR [20]. The decrease in mtDNA copy number detected by us can be considered an adaptive mechanism for the “soft-separation” of the electron transport chain, which also protects cells from the vicious circle of ROS production. Reducing the level of ROS by suppressing its production or stimulating utilization is a promising approach in the treatment of IR [20].

Mitochondria are involved in the regulation of carbohydrate metabolism and contribute to the pathogenesis of T2DM in obesity [21]. We detected high plasma TNF-α level and mtDNA copy numbers in different tissues in patients with T2DM with respect to those in obese patients without T2DM. mtDNA copy numbers in the GO (grade II and grade III) and SAT (grade III) in obese patients with T2DM significantly exceeded those in patients without T2DM. A positive correlation of mtDNA copy numbers in the GO and SAT with serum glucose level was found in T2DM patients with morbid obesity (BMI > 40 kg/m^2^), and a negative correlation of mtDNA copy numbers in the GO with glucose was found in patients without T2DM (with grade I obesity), which, in our opinion, helps maintain normoglycemia (Fig. 1t, u, v). Based on these findings, we postulate a positive effect of reducing mtDNA in AT on carbohydrate metabolism in morbid obesity, which may help maintain glucose levels within reference values.

An increase in the mass of VATs is associated with an increased inflammatory background and development of IR [22], and the role of SATs is not completely determined. The change in SAT metabolism in obesity may be protective in nature with respect to IR and its complications [8, 23]. We observed positive correlations between the amount of mtDNA and expression level of the *ADIPOQ* gene (Fig. 1s) in the SAT in patients without T2DM (with grade I obesity), which was confirmed in the study data. In this case, an increase in the production of adiponectin can suppress the production of TNF-α in this type of fat depot [23].

With respect to the homeostasis of glucose, we have demonstrated the dual role of chemerin in obesity: positive - with obesity and no disruptions in carbohydrate metabolism and negative - with obesity associated with T2DM. The location of chemerin synthesis plays a key role in metabolism [24]. Chemerin is an adipokine regulating the processes of lipogenesis, metabolism and inflammation through the activation of chemokine-like receptor-1 (CMKLR1) [8]. Other receptors for the ligand are CMKLR1 or Chem23, GPR1 and CCRL2 [25]. Some studies indicate the proinflammatory properties of chemerin [26–28]. Сhemerin affects the production of proinflammatory mediators (TNF-α, IL-1β, and IL-6), which leads to the activation of the MAPK pathway and suppression of insulin signal transmission in human skeletal muscle cells [29, 30]. In patients with BMI < 40 kg/m^2^, we observed positive correlations between plasma chemerin level and *TNF-α* gene expression in the GO, Mes and SAT (Fig. 1h, i, j).

TNF-α can also affect the production of chemerin in differentiated adipocytes of the 3 T3-L1 line [31, 32]. In patients with BMI < 40 kg/m^2^, the plasma level of TNF-α was positively correlated with the expression of the *RARRES2* gene in the Mes (Fig. 1e), suggesting that these mediators are mutually regulated. In the patients without T2DM (with grade I and II obesity), the relative expression levels of *TNF-α* and *RARRES2* genes in the GO were correlated (Fig. 1f, g).

Chemerin increases the secretion of proinflammatory and prodiabetic adipokines, with negative systemic effects on proinflammatory adipokines [26, 27]. Subclinical inflammation in AT is systemic in morbid obesity, which can be confirmed by positive associations of the plasma level of chemerin with CRP in patients with T2DM (*r* = 0.76, *p* < 0.01) (Fig. 1d).

In contrast, chemerin can prevent TNF/TNFA activation by inhibiting the NF-kB and CRK/p38 signaling pathways [8] by binding to the CMKLR1 receptor [8]. Under physiological conditions, chemerin may play anti-inflammatory roles within the visceral fluid. This was confirmed by the positive correlations between the expression of *ADIPOQ* and *RARRES2* genes in the GO in the control group (Fig. 1k).

The level of chemerin expression in the GO and SAT increased in patients with T2DM and did not change in patients without T2DM relative to that in control individuals. In contrast, the plasma level of chemerin increased mainly in obese patients without T2DM. Given the presence of several isoforms of chemerin with both pro- and anti-inflammatory activity, it is logical to assume that the production of proinflammatory forms is mainly prevalent in obese patients with T2DM, whereas anti-inflammatory forms are likely synthesized in other locations.

In turn, the activation of CMKLR1 exerts negative effects on mitochondrial biogenesis and division [8]. The plasma level of chemerin was negatively correlated with mtDNA copy number in MNCs (Fig. 1w) and positively correlated with BMI in pre-obese individuals (Fig. 1c). A reciprocal link between mtDNA duplication and chimeric plasma level was also observed. The plasma level of chemerin was negatively correlated with mtDNA copy number in the Mes in patients without T2DM (with grade III obesity) (Fig. 1o). In addition, in all obese patients without T2DM, an increase in the plasma level of chemerin and its expression in biopsy samples of the Mes was recorded.

Therefore, it can be speculated that chemerin regulates local mtDNA copy number in the GO and Mes in cases of morbid obesity. In biopsy specimens of the prostate, mtDNA copy number was negatively associated with the expression of the *RARRES2* gene in patients with morbid obesity without T2DM (Fig. 1x, y). A negative relationship was observed between mtDNA copy number in biopsy specimens of the GO and plasma chemerin and chemerin expression in the GO. Thus, in patients with morbid obesity without T2DM, chemerin may exert a protective effect against the development of IR by regulating the number of copies of mtDNA.

The dual role of chemerin in inflammatory processes and metabolism provides a link between chronic inflammation and energy metabolism in obesity, as well as the complications associated with these processes.

It should be noted that antioxidant protective enzymes in different tissues function with different efficacy [33, 34] and may display different responses to genotoxic agents during obesity. In this regard, it is necessary to consider the heterogeneity of the response of different tissues of the body to cytotoxic damage. Therefore, it can be assumed that the amount of mtDNA in various types of insulin-dependent tissues, such as AT and blood cells, is an important indicator of various metabolic disorders. The quantification of mtDNA in various biological samples can thus be a valid tool for predicting and evaluating the effectiveness of treatment of IR.

The obtained data on changes in copy number of mtDNA can be used to diagnose early disorders of carbohydrate metabolism in obese patients. In addition, further studies of the effect of different isomers of chimerin on mitochondrial biogenesis may contribute to the development of targeted therapy for type 2 diabetes.

## Conclusions

1. We assume the existence of a compensatory mechanism represented by an increase in mtDNA copy number under the influence of proinflammatory factors.

2. Based on the data obtained, we postulate a positive effect of reducing mtDNA copy number in morbid obesity in the absence of T2DM on carbohydrate metabolism, which can help maintain glucose levels within reference values.

3. The negative association between plasma chemerin and mtDNA copy number in the Mes, as well as between mtDNA copy number and chemerin expression in the Mes, in the group with BMI > 40 kg/m^2^ without T2DM demonstrates the protective effect of chemerin against the development of IR via the regulation of the number of copies of mtDNA in adipose tissues.

## Abbreviations

AT: Adipose tissue
ATP: Adenosine triphosphate
BMI: Body mass index
ddPCR: Droplet Digital PCR
GO: Greater omentum
IR: Insulin resistance
Mes: Mesentery of the small intestine
MNC: Blood mononuclear cells
mtDNA: Mitochondrial DNA
SAT: Subcutaneous adipose tissue
T2DM: Type 2 diabetes mellitus
